# Enhancing multiple myeloma staging: a novel cell death risk model approach

**DOI:** 10.1007/s10238-024-01337-9

**Published:** 2024-05-08

**Authors:** Zeyu Deng, Hongkai Zhu, Zhaoshun Yuan, Rong Zhang, Zhihua Wang, Heng Li, Le Yin, Xueqin Ruan, Zhao Cheng, Ruijuan Li, Hongling Peng

**Affiliations:** 1 Department of Hematology, The Second Xiangya Hospital of Central South University, Changsha, Hunan People’s Republic of China; 2 Institute of Hematology, Central South University, Changsha, Hunan People’s Republic of China; 3 Hunan Engineering Research Center of Cell Immunotherapy for Hematopoietic Malignancies, Changsha, Hunan People’s Republic of China; 4 Hunan Key Laboratory of Tumor Models and Individualized Medicine, Changsha, Hunan People’s Republic of China; 5 National Cancer Center Exploratory Oncology Research & Clinical Trial Center, Kashiwa, Japan; 6 Department of Cardiovascular Surgery, The Second Xiangya Hospital of Central South University, Changsha, Hunan People’s Republic of China

**Keywords:** Multiple myeloma, Gene expression signature, Cell-death

## Abstract

The prognostication of survival trajectories in multiple myeloma (MM) patients presents a substantial clinical challenge. Leveraging transcriptomic and clinical profiles from an expansive cohort of 2,088 MM patients, sourced from the Gene Expression Omnibus and The Cancer Genome Atlas repositories, we applied a sophisticated nested lasso regression technique to construct a prognostic model predicated on 28 gene pairings intrinsic to cell death pathways, thereby deriving a quantifiable risk stratification metric. Employing a threshold of 0.15, we dichotomized the MM samples into discrete high-risk and low-risk categories. Notably, the delineated high-risk cohort exhibited a statistically significant diminution in survival duration, a finding which consistently replicated across both training and external validation datasets. The prognostic acumen of our cell death signature was further corroborated by TIME ROC analyses, with the model demonstrating robust performance, evidenced by AUC metrics consistently surpassing the 0.6 benchmark across the evaluated arrays. Further analytical rigor was applied through multivariate COX regression analyses, which ratified the cell death risk model as an independent prognostic determinant. In an innovative stratagem, we amalgamated this risk stratification with the established International Staging System (ISS), culminating in the genesis of a novel, refined ISS categorization. This tripartite classification system was subjected to comparative analysis against extant prognostic models, whereupon it manifested superior predictive precision, as reflected by an elevated C-index. In summation, our endeavors have yielded a clinically viable gene pairing model predicated on cellular mortality, which, when synthesized with the ISS, engenders an augmented prognostic tool that exhibits pronounced predictive prowess in the context of multiple myeloma.

**Supplementary Information:**

The online version contains supplementary material available at 10.1007/s10238-024-01337-9.

## Introduction

Multiple myeloma is characterized b﻿y malignant plasma monoclonal in the bone marrow with an incidence of 6–7 per 100,000 humans a year globally [1, 2]. Biomarkers foreseeing prognosis is essential for the distinguishment of high-risk MM patients and the development of personalized curation. From the 1960s to 1990s, a spectrum of clinical and laboratory parameters independently related to prognosis was found, including hemoglobin level, serum calcium, serum creatinine, the severity of bone lesions, serum beta2 microglobulin (Sβ2M), serum levels of C-reactive protein and albumin [3–7]. Some stage scoring systems based on clinical parameters emerged, including DC and ISS. Compared with DC, ISS shows stronger availability and efficiency in distinguishing high-risk groups [8]. Subsequently, the extensive application of interphase FISH tech revealed a series of abnormal karyotypes influencing diagnosis or prognosis, such as Trisomies, t(11;14), t(6;14), Monosomy 13, 1q gain, 1p del, MYC 8q24, t(4;14), 17p del, t(14;16) and t(14;20)[9]. As a result, R-ISS regrouped MM patients by adding LDH and three abnormal karyotypes (del(17p), t(4;14) and translocation t(14;16)) to upgrade ISS [10]. Considering that serum beta2 microglobulin and LDH level may be affected by comorbidities, chromosomal abnormalities existing meet the requirement of the criterion of R-ISS stage 3 regardless of LDH or beta2 microglobulin. However, even if R-ISS is an ameliorated risk stratification method with increasing predictive ability compared with ISS, abnormal karyotypes are hard to illustrate the heterogeneity between patients. As the discovery of new agents would overcome the risk brought by a certain karyotype, the ability of RISS to identify high-risk MM patients may gradually weaken. A predictive algorithm utilizing gene expression profile (GEP) has exhibited its flexibility. Gene expression profiles can take the integration of multiple gene abnormalities into several gene pathways [11]. Prior studies have explored the prognostic utility of integrating transcriptomic models with the International Staging System (ISS) to predict outcomes in multiple myeloma (MM). For instance, the SKY92, a signature of 92 genes identified from the HOVON65/GMMG-HD4 clinical trial using the Affymetrix GeneChip Human Genome U133 Plus 2.0 Array, has been shown to enhance the prognostic accuracy for MM patients when combined with the ISS, across different disease stages and treatment regimens [10, 12, 13]. The principal limitation of transcriptomic models in clinical application is the absence of absolute quantification in gene expression, regardless of whether next-generation sequencing or microarray technologies are used. Additionally, the presence of batch effects complicates the generalization of transcriptomic models across different platforms. Gene pairing presents an effective strategy to circumvent these issues. By focusing on the relative expression levels between two genes, gene pairing significantly mitigates the impact of batch effects. W. Kong and colleagues have developed a prognostic model based on gene pairing using pyroptosis and immune-related genes, which has demonstrated markedly enhanced clinical applicability compared to models that do not utilize gene pairing [14].

The disability of cell death is direct cause of human cancer developing and enhancing drug resistance. Mutations of Genes controlling programmed cell death can lead to gene instability through passage accumulation and neoplasia [15]. Apoptosis [16–18], autophagy [19] and pyroptosis [20, 21] were recognized as programmed cell death processes via certain molecular mechanisms. Although it is still uncertain whether ferroptosis is a physiological process formed under the long-term evolution of cells, ferroptosis has been reported as a tumor suppression process, especially metastasis-prone and drug-resistant cancer cells under the mesenchymal situation exhibit more sensitivity to ferroptosis [22]. This study culminates in the creation of a novel gene-pairing-based prognostic model, utilizing an aggregated cell death gene repository. This model is adeptly integrated with the established International Staging System (ISS) scoring framework, resulting in a refined and improved ISS. The resultant model demonstrates superior prognostic performance, heralding a significant advancement in the predictive accuracy of patient outcomes.

## Methods

### Data collection

For cell death signature construction, gene expression profiles of CD138^+^ selected plasma cell and clinical information from five MM cohorts with a total of 2080 MM patients were utilized in our study. Among them, GSE136337 (GPL27143), GSE57317 (GPL570)、GSE24080 (GPL570) and GSE19784 (GPL570) are microarray data obtained from the Gene Expression Omnibus (GEO) database (http://www.ncbi.nlm.nih.gov/geo/). Microarray gene expression data were undergo normalized between arrays before further analysis. The MMRF-CoMMpass dataset containing level three RNA sequencing (RNA-seq) FPKM data was acquired in The Cancer Genome Atlas (TCGA) website (https://portal.gdc.cancer.gov/repository). The detailed clinic-pathological information for each cohort is represented in Table 1. Apoptosis and pyroptosis related genes were downloaded from Molecular Signatures Database v7.5.1 (https://www.gsea-msigdb.org/gsea/msigdb/index.jsp). FerrDb (http://www.zhounan.org/ferrdb/legacy/index.html) and HADb (http://www.autophagy.lu/index.html) were retrieved to get ferroptosis and autophagy associated genes (Supplementary Table 1). Four groups of genes were integrated for the next step of the analysis.

**Table 1:** Clinical indices of the training and validation cohorts

Characteristics	Training set MMRF-CoMMpass (*n* = 796)	Validation set GSE136337 (*n* = 415)	Validation set GSE24080 (*n* = 558)	Validation set GSE57317 (*n* = 55)	Validation set GSE19784 (*n* = 256)
*Gender*					
female	328 (41%)	158 (38%)	221 (40%)	–	–
male	468 (59%)	257 (62%)	337 (60%)	–	–
*Age*					
≤ 65 years	430 (54%)	299 (72%)	431 (77%)	–	–
> 65 years	366 (46%)	116 (28%)	127 (23%)	–	–
*ISS*					
I	260 (33%)	163 (39%)	295 (53%)	–	109 (43%)
II	286 (36%)	133 (32%)	144 (26%)	–	70 (27%)
III	250 (31%)	119 (29%)	119 (21%)	–	77 (30%)
*R-ISS*					
I	–	149 (36%)	–	–	–
II	–	243 (59%)	–	–	–
III	–	23 (5%)	–	–	–
*albumin*					
≥ 3.5 g/dL	–	331 (80%)	481 (86%)	–	–
< 3.5 g/dL	–	84 (20%)	77 (14%)	–	–
*β2M*					
< 3.5 mg/L	–	187 (45%)	319 (57%)	–	–
3.5–5.5 mg/L	–	109 (26%)	120 (22%)	–	–
≥ 5.5 mg/L	–	119 (29%)	119 (21%)	–	–
*LDH*					
≤ 250 U/L	–	392 (94%)	508 (91%)	–	–
> 250 U/L	–	23 (6%)	50 (9%)	–	–
*Survival state*					
Alive	605 (76%)	239 (58%)	386 (69%)	43 (78%)	166 (65%)

β2M β2-microglobulin, LDH lactate dehydrogenase

### Construction and validation of a cell-death risk score

We utilized the MMRF-CoMMpass array, the largest cohort within our study (*n* = 796), to establish a training set. Two-thirds of these samples were randomly selected for model training, with the remaining one-third reserved for internal validation. External validation was conducted using datasets from GSE24080, GSE136337, GSE57317, and GSE19784.

**Fig. 1:**
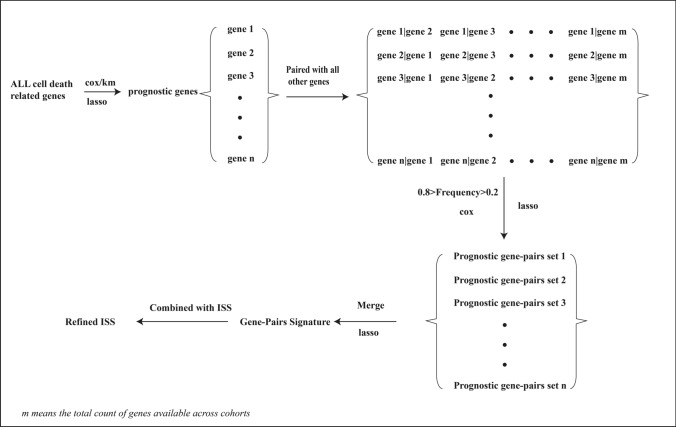
Schematic representation of the analytical workflow

Within the training cohort, univariable Cox regression and Kaplan–Meier (KM) survival analyses were employed to identify cell-death genes significantly associated with MM prognosis. An optimal cut-off threshold delineated patients into two comparative groups for KM curve analysis. Genes meeting a *p* value criterion of less than 0.01 in Cox or KM analyses were advanced for further evaluation. The LASSO method was then utilized to pinpoint genes most prognostically salient, incorporating a penalty coefficient to refine this selection [23]. Following identification, each prognostic gene was paired with every other gene, assigning a binary outcome based on their relative expression. This was done such that if gene A's expression level was higher than gene B’s, the gene pair A|B was assigned a value of 1, and conversely, a value of -1 if lower. To preserve variability and mitigate batch effects, gene pairs with a frequency greater than 80% or less than 20% of the total sample size were excluded. Subsequently, univariate Cox regression was applied to each gene pair, and those with a false discovery rate (FDR) less than 0.05 were retained for additional LASSO regression refinement. The culmination of this process was the assembly of a Cox LASSO regression-based prognostic model of cell-death gene pairs, details of which are depicted in Fig. 1.

The resultant model equation allowed for calculation of individual MM patient risk scores, given by RiskScore = ∑(coef*GenePAIR). In the training cohort, the 'survminer' package's surv_cutpoint function was employed to ascertain the optimal cut-off, thus maximizing the survival discrepancy between high- and low-risk groups. This cut-off was uniformly applied to the validation cohorts to stratify risk.

### Combining ISS with cell-death signature

Our initial multivariate Cox regression analyses incorporated the ISS and other clinical indicators with the cell-death pairing model across multiple arrays to determine if the cell-death model functioned as an independent prognostic factor.

Given that all arrays, with the exception of the smaller GSE57317 cohort, included ISS scoring data, we sought to amalgamate the cell-death pairing model with ISS in a larger sample setting, thereby devising a modified ISS. This integration produced six potential groupings based on cell-death risk (high or low) and ISS scores (1, 2, or 3), effectively stratifying MM patients into six categories. Based on the collective KM curves, patients were reclassified into three distinct risk categories (high, intermediate, and low). The predictive performance of the modified ISS was gauged against other models using the c-index.

### Statistical analysis

All statistical analyses in this study were performed using R software (version 4.0.5). The 'survival' and 'survminer' packages facilitated Kaplan–Meier survival curve comparisons and Cox regression analyses. The 'glmnet' package was used for Lasso Cox regression, while the 'timeROC' package was employed for depicting ROC curves and calculating the AUC for prognostic accuracy. The 'meta' package integrated the c-index effect sizes from multiple arrays using a random effects model. The log-rank test calculated *p* values for survival analysis, with a significance threshold set at *p* < 0.05.

## Results

### Construction of the cell-death signature

**Fig. 2:**
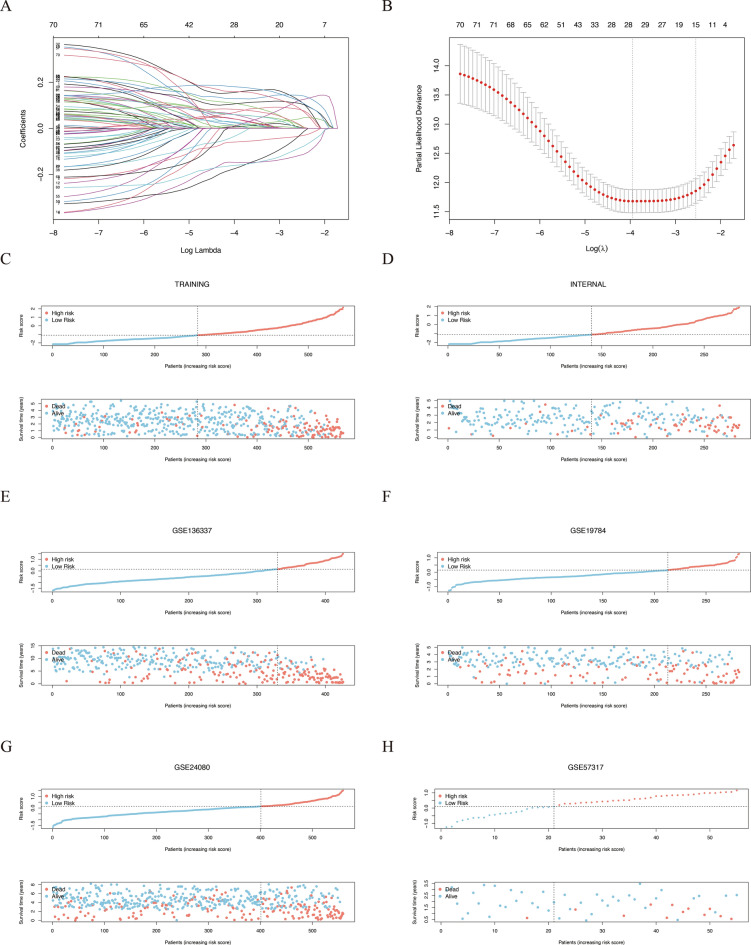
Formulation of the cell-death gene-pairing model. **A**-**B** During LASSO regression, escalating penalty coefficients nullify many gene pairs' coefficients, while concomitantly the partial likelihood variation diminishes, achieving a nadir with a refined set of 28 gene pairs. **C**-**H** Distribution of risk scores and associated trends in survival status and duration among MM samples with increasing risk scores. Designations of the respective arrays are indicated at the top of each panel

In our investigation, a cohort of 2080 patients with multiple myeloma (MM) and associated survival data was assembled for the training and validation of prognostic models. The MMRF-CoMMpass dataset, with its robust sample size, was designated as the training set. A subset of 1145 genes associated with cell death and correlated with survival outcomes was identified through univariate Cox regression and Kaplan–Meier survival analyses. A nested lasso regression approach was employed to refine this gene set. This process involved the selection of a penalty coefficient at the minimum partial likelihood deviation plus one standard deviation, optimizing the model's fit. This refined our prognostic gene set down to nine key candidates (CD38, DLGAP5, ELOVL6, FLNA, HMGB3, LMNB1, LTBP1, MCM4, PFDN2). These genes were then paired with 11,769 others to construct a novel gene pairing matrix. In this matrix, a gene pair such as CD38|TP53 would be assigned a value of 1 if CD38's expression exceeded that of TP53, and -1 otherwise. We rigorously screened these gene pairs, excluding those with minimal variability and retaining those with prognostic relevance as indicated by a false discovery rate (FDR) below 0.01, followed by additional refinement via lasso regression. This process culminated in a prognostic model consisting of 28 gene pairs (Fig. 2a-b), detailed in Supplementary Table 2. The methodology for risk score calculation is detailed in the Methods section. Within the training cohort, an optimal cut-off point of 0.15 differentiated high-risk patients—those with risk scores above this threshold—from the low-risk cohort. This cut-off was consistently applied across internal and external validation sets (Fig. 2c-h).

**Fig. 3:**
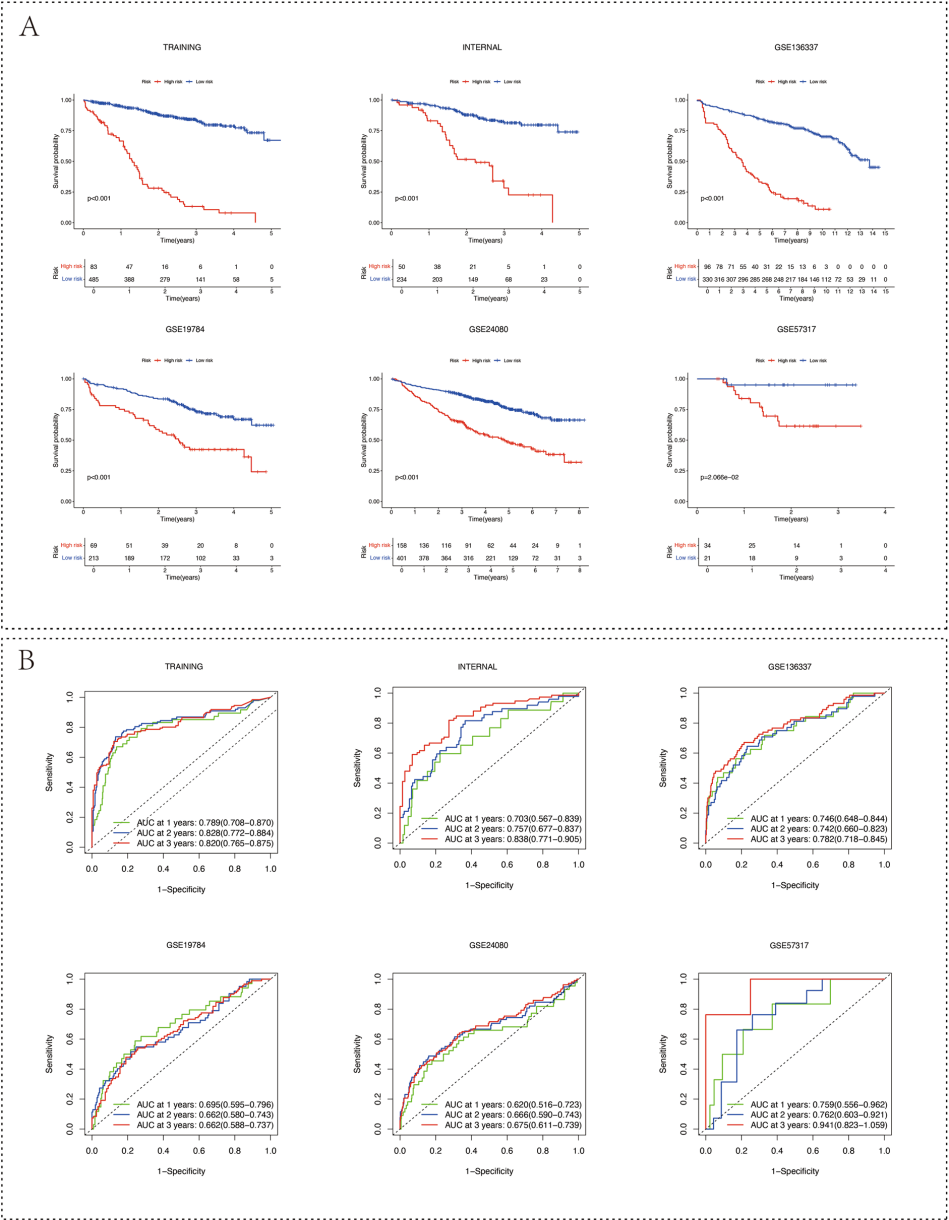
Kaplan–Meier survival plots and Time-dependent ROC curves. **A** These six Kaplan–Meier curves represent the differential overall survival between high- and low-risk groups stratified by the cell-death pairing model across various datasets. High-risk groups are depicted by red curves, while low-risk groups are represented in blue. **B** Time-dependent ROC curves assess the predictive efficacy of the cell-death risk scores, with each curve corresponding to 1-, 2-, and 3-year overall survival predictions. The specific arrays for each graph are denoted at the top of the visuals

We assessed the impact of risk stratification on overall survival, revealing that high-risk patients exhibited significantly reduced survival across training and validation cohorts (Fig. 3a). The predictive capacity of the cell-death risk score, particularly its accuracy for 1-, 2-, and 3-year survival predictions, was impressive across all datasets, with area under the curve (AUC) metrics exceeding 0.6 (Fig. 3b).

**Fig. 4:**
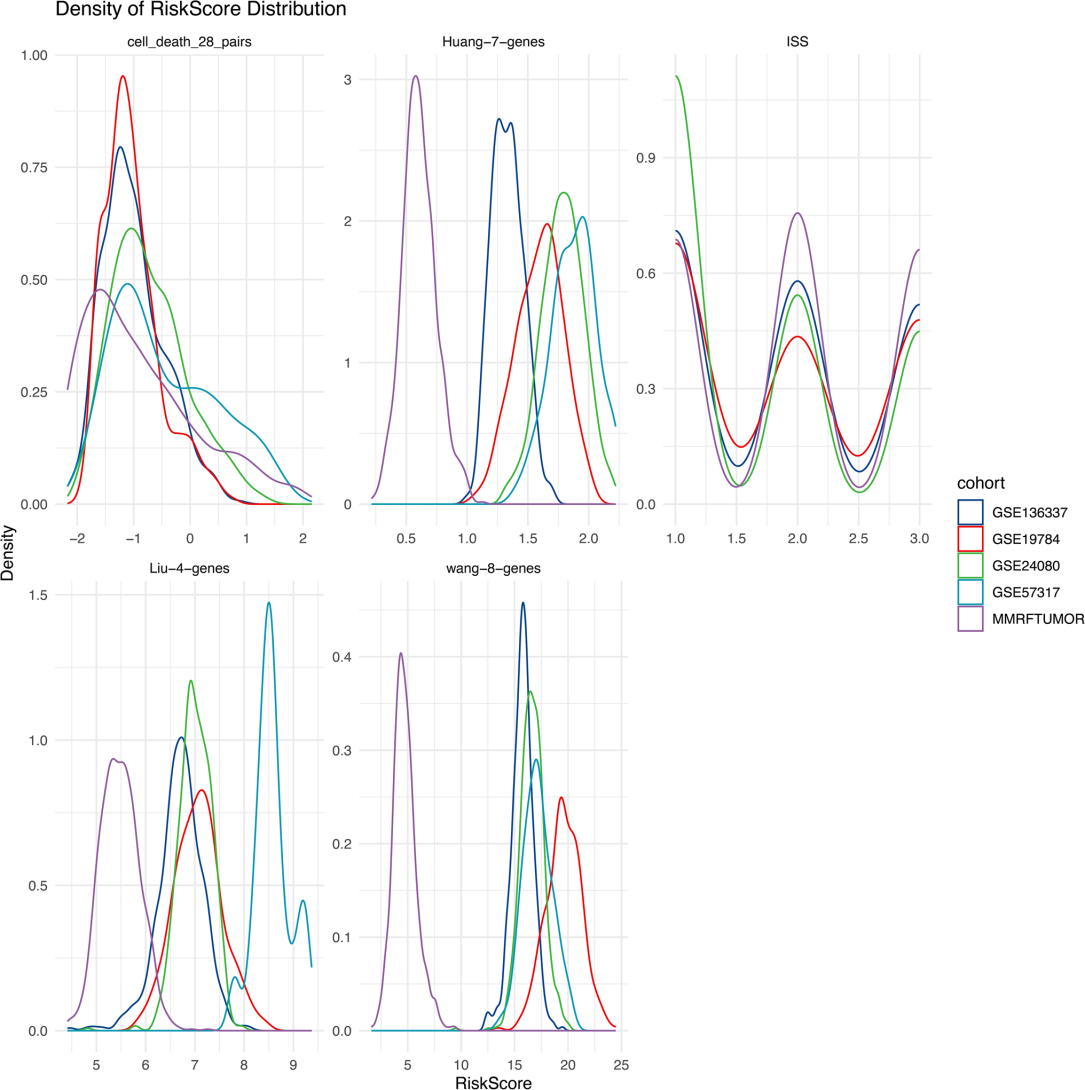
Density distributions of risk scores for disparate models. This illustration is segmented into five divisions, each correlating with outcomes from different models, identified at the head of each segment. The 'cell_death_28_pairs' demarcates the model investigated in this study. Variegated colors denote distinct arrays

One of the most significant challenges in the adoption of gene expression-based prognostic models in a clinical setting is their susceptibility to batch effects, which undermine score stability, and discrepancies arising from different technological platforms, such as next-generation sequencing versus microarrays. Our gene-pairing strategy addresses these challenges effectively. When we juxtaposed our 28-gene-pairs cell-death model with three other gene expression models and the International Staging System (ISS) risk scores, our model demonstrated a high degree of consistency across the five datasets, contrary to the other models [24–26] (Fig. 4). The minimal variation in ISS score distributions across the datasets suggests that the variability in gene expression model scores may predominantly be attributed to batch effects or platform differences, highlighting the greater clinical utility of our paired model.

### The cell-death pairing model as an independent prognostic indicator

**Fig. 5:**
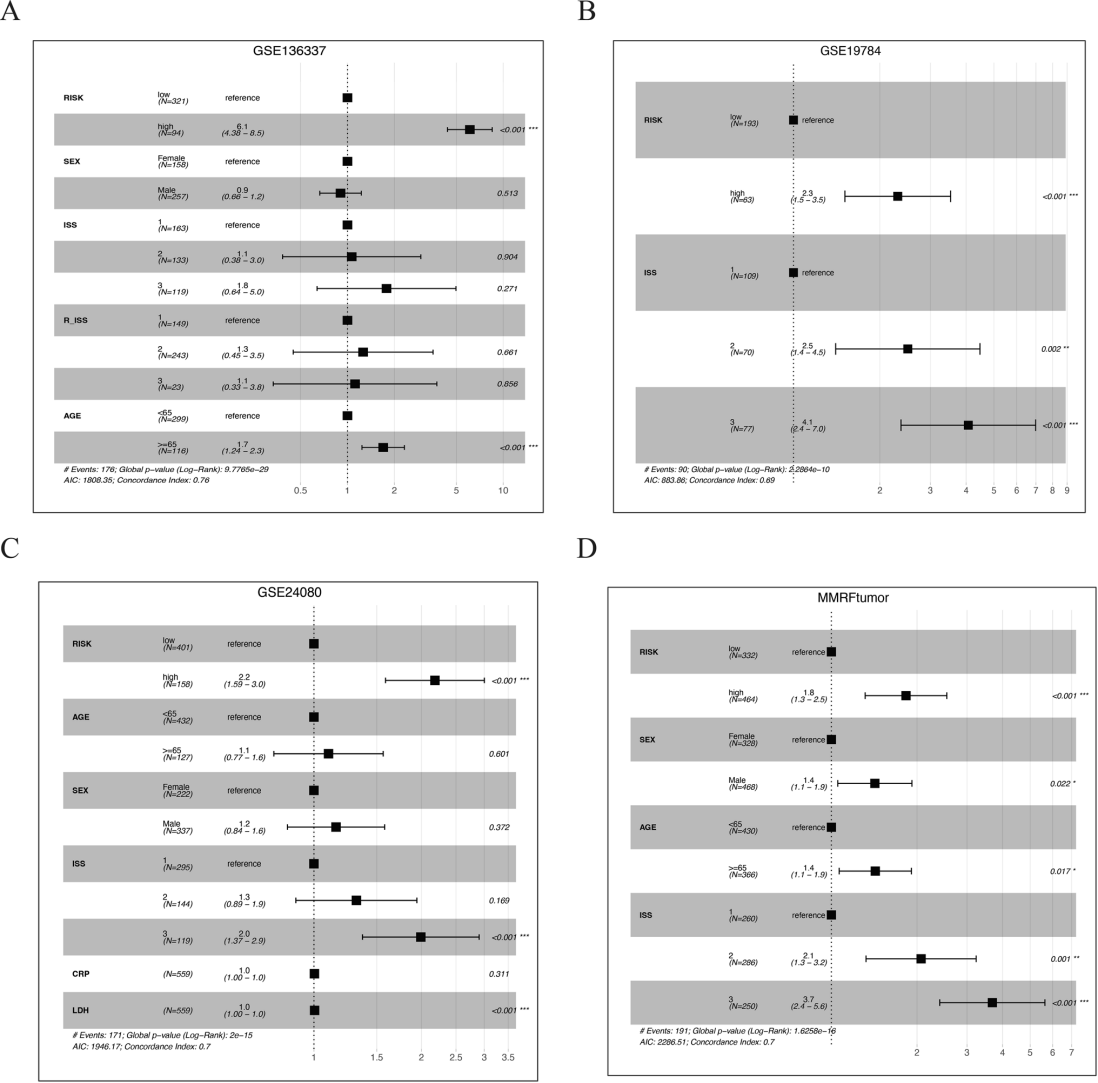
Cell-death risk model as an autonomous prognostic determinant. Forest plots in panels A-D correspond to multivariate COX regression analyses across four different arrays, each specified at the apex of the plot. 'RISK' refers to the cell-death risk model within the first column of the forest plot, where, in all scenarios, the high-risk group exhibits a Hazard Ratio (HR) significantly in excess of unity relative to the low-risk group

We conducted a multivariate Cox regression analysis integrating the cell-death risk score with other clinical indices, with careful consideration of the varying completeness of clinical data across datasets. GSE57317 was excluded due to insufficient clinical data. For the four datasets with comprehensive clinical data (GSE136337, GSE19784, GSE24080, MMRFtumor), even after adjusting for variables such as age, gender, and ISS stage, the high-risk group continued to demonstrate significantly inferior prognostic outcomes compared to the low-risk group (Fig. 5). Hence, the cell-death risk score emerged as an independent prognostic indicator.

### Integrating ISS with the cell-death model

**Fig. 6:**
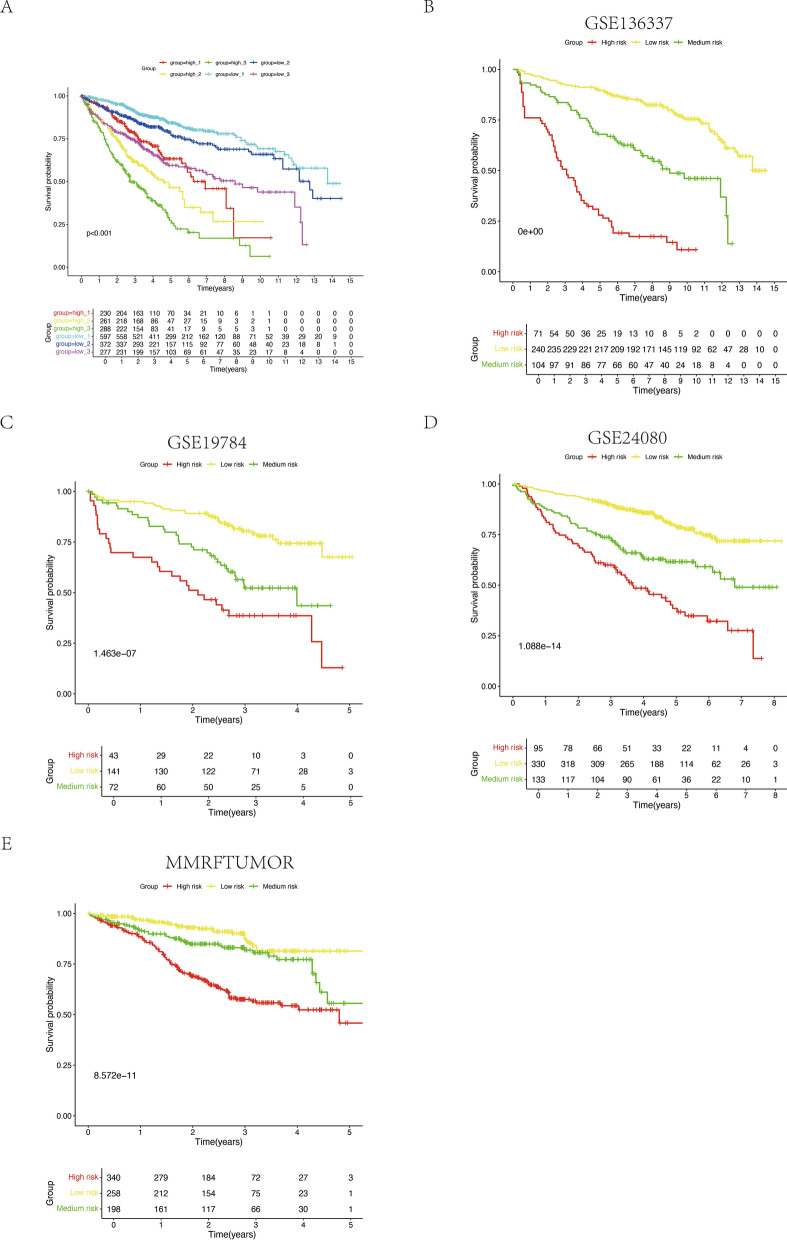
Synthesis of the cell-death risk model with ISS to establish refined new ISS categorizations. **A** An overarching survival comparison across six MM sample cohorts within the amalgamated array. **B**-**E** Survival contrasts among refined new ISS designations across four discrete arrays

With ISS data available in four datasets (GSE136337, GSE19784, GSE24080, MMRFtumor), covering a total of 2026 samples, we stratified MM patients into six novel categories based on ISS and cell-death risk scores. The Kaplan–Meier survival curves of these categories revealed similar prognoses within certain groups, prompting a reclassification into three risk categories: a low-risk group (combining low_1 and low_2), an intermediate-risk group (high_1 and low_3), and a high-risk group (high_2 and high_3), herein termed the Refined new ISS (Fig. 6a; Table 2). This new stratification showed a significant gradation in overall survival across the risk categories, which was statistically significant (Figs. 6b-e).

**Table 2:** Refined new ISS Re-categorization based on cell death risk and ISS

Cell death risk group	ISS	Refined new ISS
low	1	low
low	2	low
high	1	medium
low	3	medium
high	2	high
high	3	high

**Fig. 7:**
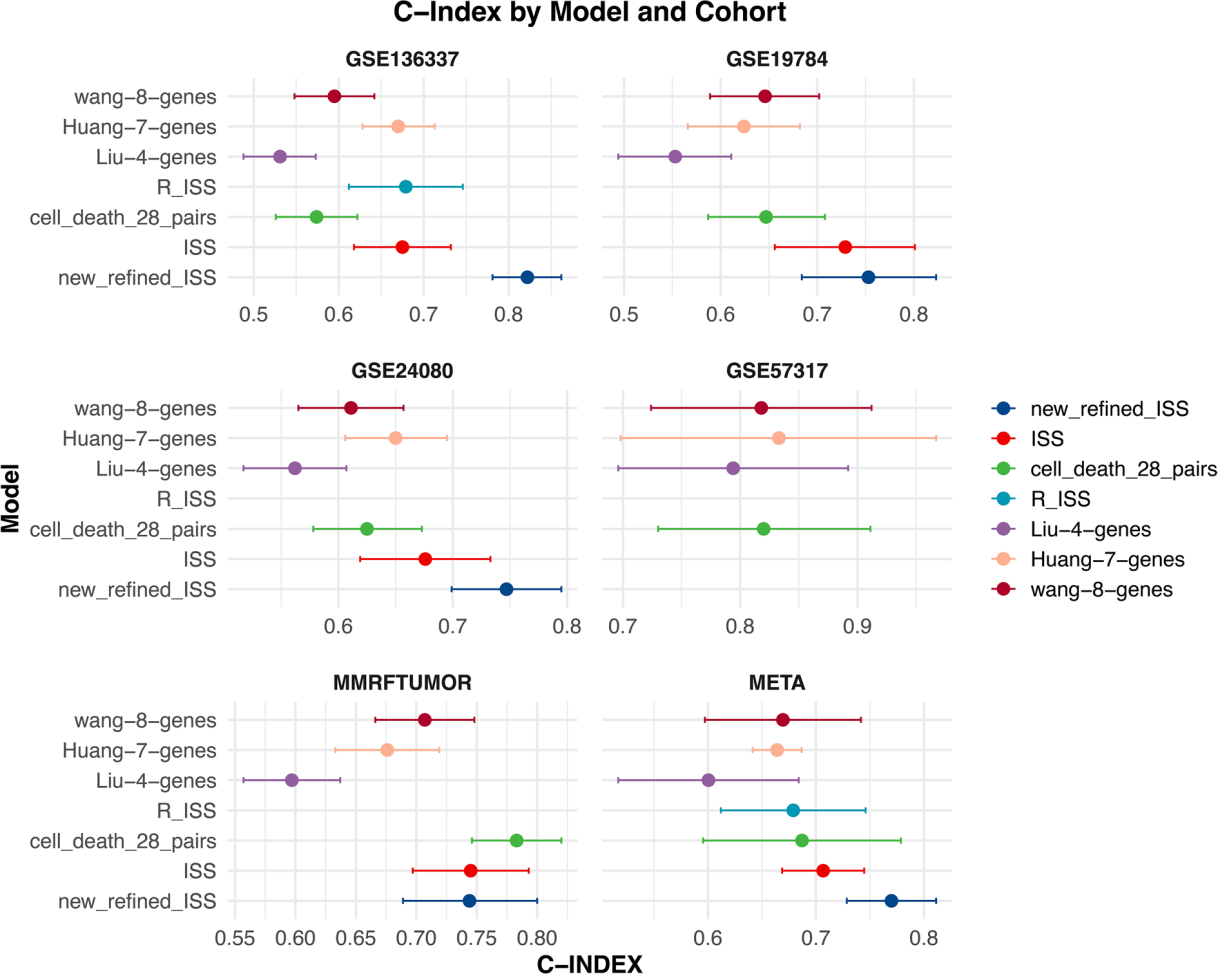
Prognostic performance evaluation across models. The C-INDEX is harnessed to appraise predictive performances, with the forest plot elucidating the 95% confidence intervals of the indices. The diagram is partitioned into six sections, the initial five pertain to individual arrays, while the concluding segment encapsulates a META analysis, synthesizing findings from the collective arrays. The new_refined_ISS is distinguished by its optimal predictive prowess

The prognostic performance of the Refined new ISS was assessed using the concordance index (C-INDEX) and compared against other models. The Refined new ISS outperformed all other models in multiple datasets, with its C-INDEX being significantly superior to both R-ISS and ISS in dataset GSE136337 that included R-ISS data (Fig. 7). A meta-analysis using a random effects model, which accounted for the multiplicity of datasets, further confirmed the superior performance of the Refined new ISS (Fig. 7, META section lower right). These findings underscore the enhanced prognostic accuracy of the Refined new ISS over the conventional ISS or R-ISS.

## Discussion

In our research, we have developed a robust prognostic model comprising 28 gene pairs, employing a sophisticated nested lasso methodology. Utilizing a standardized cut-off value of 0.15, we achieved a precise delineation of high- and low-risk groups. Integration with the International Staging System (ISS) facilitated a reclassification of multiple myeloma (MM) patients into distinct risk categories: low, intermediate, and high. The prognostic capacity of the ISS was notably enhanced by the incorporation of the cell-death risk model, yielding superior predictive power. This study contributes a clinically applicable gene-pairing model that augments the prognostic precision of the ISS. However, the exclusive presence of R-ISS data within the GSE136337 dataset constrains our capacity to demonstrate the superiority of our refined ISS over R-ISS, as validation is limited to this single dataset. Therefore, a broader scope of multicentric data is essential to further substantiate our model's superior capabilities. With the diminishing costs and increasing clinical adoption of second-generation sequencing for personalized diagnosis and treatment, our gene-pairing model—characterized by its reduced sensitivity to batch effects—holds promising potential for clinical implementation and may significantly enhance prognostic outcomes in MM patients.

## Supplementary Information

Below is the link to the electronic supplementary material.

Supplementary file1 (XLSX 57 KB)

## Data Availability

Data sharing is not applicable to this article as no datasets were generated or analyzed during the current study.
